# “What if I get sick, where shall I go?”: a qualitative investigation of healthcare engagement among young gay and bisexual men in Nairobi, Kenya

**DOI:** 10.1186/s12889-023-17555-x

**Published:** 2024-01-02

**Authors:** Samuel Waweru Mwaniki, Peter Mwenda Kaberia, Peter Mwangi Mugo, Thesla Palanee-Phillips

**Affiliations:** 1 School of Clinical Medicine, Faculty of Health Sciences, University of the Witwatersrand, Johannesburg, South Africa; 2 Department of Health Services, Administration and Campus Support Services, University of Nairobi, Nairobi, Kenya; 3 Department of Mathematics, Faculty of Science and Technology, University of Nairobi, Nairobi, Kenya; 4 Kenya Medical Research Institute - Wellcome Trust Research Programme, Nairobi, Kenya; 5 Wits Reproductive Health and HIV Institute, Faculty of Health Sciences, University of the Witwatersrand, Johannesburg, South Africa; 6 Department of Epidemiology, School of Public Health, University of Washington, Seattle, WA USA

**Keywords:** Andersen’s model, Healthcare providers, HIV, Mental health, Sexually transmitted infections, Stigma and discrimination, Telehealth, Young men who have sex with men

## Abstract

**Background:**

Globally, young gay, bisexual and other men who have sex with men (YMSM) experience a disproportionate burden of disease compared to young heterosexual men and older MSM. However, YMSM experience major inequities in access and use of health services. We sought to gain a detailed understanding of YMSM’s healthcare engagement experiences across public, private, tertiary institution-based and MSM-friendly health facilities in Nairobi, Kenya, to inform development of interventions to improve access and use of health services by YMSM.

**Methods:**

In September 2021, in-person qualitative in-depths interviews were conducted among 22 YMSM purposively sampled from 248 YMSM who had previously participated in a respondent-driven sampling integrated bio-behavioral survey. Interviews were done in English, transcribed verbatim and analyzed descriptively using NVivo version 12.

**Results:**

Participants were 18–24 years old, all identified as cisgender male, three-quarters as gay and a quarter as bisexual. Themes that were defined from the analysis included: YMSM’s experiences during healthcare seeking in various clinical settings, priority health needs, desired healthcare provider (HCP) characteristics, and the potential role of digital health interventions in improving access and use of health services. Participants relayed experiences of prejudice, stigma and discrimination when seeking services in public and institution-based health facilities, unlike in community pharmacies, private and MSM-friendly health facilities where they felt they were handled with dignity. Health needs prioritized by YMSM centered on prevention and control of HIV, sexually transmitted infections (STIs), depression and substance abuse. Participants desired HCPs who were empathetic, non-judgmental and knowledgeable about their unique health needs such as management of anorectal STIs. Participants highlighted the usefulness of digital media in offering telehealth consultations and health education on subjects such as HIV/STIs prevention.

**Conclusion:**

During engagement with healthcare, YMSM experience various barriers that may cause them to postpone or avoid seeking care hence resulting in poor health outcomes. There is need to equip HCPs with knowledge, skills and cultural competencies to enable them offer equitable services to YMSM. Considerations should also be made for use of digital health interventions that may help YMSM circumvent some of the aforementioned barriers to service access and use.

## Introduction

Globally, gay, bisexual and other men who have sex with men (referred to here as MSM) experience a disproportionate burden of disease compared to men in the general population. These include but are not limited to: human immuno-deficiency virus (HIV) [1], sexually transmitted infections (STIs) [2] and mental health conditions such as anxiety, depression, suicidal ideation and substance abuse [3]. Among MSM, the subgroup of young MSM (YMSM) aged 18–24 years, experience comparatively higher disease burdens than young heterosexual men and older MSM [4]. For instance, our previous work among Kenyan YMSM found an estimated HIV prevalence of 3.6% which is six times higher than that of Kenyan heterosexual men aged 18–24 years [5]. We also observed pervasive prevalence of STIs, with more than half (58.8%) of study participants testing positive for at least one of five curable STIs (chlamydia, mycoplasma, gonorrhea, trichomoniasis or syphilis) [6]. Despite the clear need for reliable services, major inequities persist in access to HIV/STIs prevention, care, and treatment [7], and mental health [8] services for MSM, with YMSM reporting significantly lower levels of access to MSM-focused HIV prevention programs, low-cost STI testing and treatment, and mental health services [9]. Healthcare-related stigma and discrimination are major drivers of these disparities [10], especially in countries such as Kenya where same-sex practices between men are criminalized [11] and MSM are at increased risk of experiencing violence from the community [12].

Sexual stigma which is defined as negative regard, inferior status, and relative powerlessness that society collectively accords to any non-heterosexual behavior, identity, relationship, or community [13], may contribute to health disparities for MSM. Fear of judgment, condescension and other expressions of condemnation from HCPs resulting from sexual stigma, are known to deter YMSM from actively and consistently seeking services [14]. Additionally, YMSM who do not feel comfortable disclosing their same-sex practices to HCPs for fear of stigmatization, are less motivated to seek health services especially if they feel that they must hide or otherwise disguise some part of their sexual orientation, identity and/or behavior during these encounters with HCPs [15]. Discrimination, which is closely related to stigma, is the practice of treating a particular group in a society less fairly than others [16], worsens these health inequities. A global survey of YMSM found that discrimination by HCPs deterred YMSM from seeking HIV/STIs testing, counseling and treatment [17]. Results from a study at a South African university indicated that due to institutionalized stigma and discrimination, student MSM found it difficult to access services such as post-exposure prophylaxis from the health facilities within the university [18].

Despite criminalization of same-sex practices in Kenya, there is a dedicated national program within the ministry of health that leads the HIV/STIs response among key populations, including MSM [19]. Under this program, MSM access services mainly from MSM-friendly clinics which are run by community-based or non-governmental organizations [20]. Kenyan MSM have previously reported experiences of stigma and discrimination in the hands of HCPs when seeking sexual health services from public health facilities [21–23]. Tertiary institutions within the Nairobi metropolis have health facilities that provide care to their student population [24–26]. A study among HCPs drawn from some of the tertiary institution-based health facilities, demonstrated that HCPs were cognizant of their professional duty to care for YMSM [27]. However, some of the actions by HCPs such as attempts to convert YMSM to the socially acceptable heterosexual orientation [27], may have communicated hostility toward YMSM, possibly leading the latter to postpone or avoid seeking care when they needed it.

Besides focusing on public health facilities, the experiences of MSM in healthcare settings in Kenya have not been disaggregated by age groups, despite the aforementioned particular vulnerabilities faced by YMSM. We sought to fill this gap in literature by conducting a qualitative study with YMSM from tertiary institutions to understand their experiences with services offered across public, private, institution-based and MSM-friendly health facilities in Nairobi, Kenya. Findings from this study can be used to tailor interventions aimed at reducing stigma and discrimination toward YMSM in various healthcare settings, with a view to expanding YMSM’s access and use of health services.

## Methods

Study methods are summarized in the published study protocol [28], and detailed below:

### Aim

The aim of the study was to investigate experiences of YMSM during care seeking in public, private, MSM-friendly and institution-based health facilities within the Nairobi metropolis, Kenya.

### Study design and theoretical framework

This was a qualitative study in which data was collected through in-depth interviews with YMSM. Data collection, analysis and interpretation was guided by Andersen’s behavioral model of health services use [29], with modifications for vulnerable populations [30]. Specifically, we looked at the construct of characteristics of the population at risk, that takes into account the predisposing, enabling and need factors that determine healthcare engagement. The model for vulnerable populations was chosen due to YMSM’s vulnerability to ill health that results from criminalization of same-sex practices, stigma and discrimination, homophobia and violence, power imbalances in relationships and alienation from family and friends [4].

### Setting

The study was conducted in Nairobi, the capital city of Kenya and home to approximately 150 campuses of various universities and mid-level colleges [31]. The Nairobi metropolis has more than 1,000 legally registered public and private health facilities [32], including approximately five recognized non-governmental/community-based MSM-friendly clinics [20], as well as several community pharmacies [33], where YMSM may access services.

### Participants

YMSM for this study (N = 22) were selected from a larger sample (N = 248) that had previously taken part in a quantitative respondent-driven sampling (RDS)-based integrated bio-behavioral survey, whose details are described elsewhere [5, 6, 28, 34]. YMSM were eligible for the qualitative study if they had taken part in, reported interacting with a HCP in the 12 months preceding, and tested negative for HIV during the RDS-based survey. A negative HIV test was necessary since during the current study, we also asked for YMSM’s perspectives on HIV testing, pre-exposure and post-exposure prophylaxis (not reported here). It was therefore considered unethical to inquire about these perspectives from YMSM living with HIV. Fifty-two YMSM met the set eligibility criteria, from which 25 (10% of the 248 in the RDS-based survey) were selected from each of the RDS recruitment waves, with the number of YMSM selected from each wave being proportional to the size of the wave. The selection of 25 from the eligible 52 YMSM was based on pragmatic considerations by the researchers [35]. We anticipated that due to the time difference (6 months) between the RDS-based survey and the current study, some of the YMSM may not have been within the Nairobi metropolis at the time of the current study since most tertiary institutions were on recess. This would have presented us with difficulties getting these YMSM to participate in the current study. Additionally, we noted that a similar approach of selecting a tenth of participants from a RDS-based survey to take part in subsequent qualitative interviews, had been used successfully in neighboring Tanzania, even with a smaller sample of 10 participants [36]. Subsequently, these 25 YMSM were invited for the in-depth interviews, out of which 22 took part, with the remaining three unable to participate due to time constraint.

### Data collection procedures

Demographic information was collected through a short survey administered through the REDCap digital platform (Vanderbilt University, TN, USA). A semi-structured interview guide was developed by the researchers based on the aim of the study. Following Andersen’s modified model for vulnerable populations [30], the interview guide aimed to collect data on the predisposing characteristics of YMSM, especially reflecting on the impact of same-sex practices on service use. Examples of questions included: *Do you feel that healthcare providers handle MSM clients differently from others? How does the handling of MSM clients by healthcare providers affect access to services such as treatment of STIs for MSM clients?* YMSM were also asked about their priority health needs, ideal characteristics of HCPs, and their thoughts on if technology could be of any use in promoting health services use among them. Interviews were conducted in September 2021 by the first and second authors, and at central location within Nairobi, where the RDS-based survey had previously taken place. Interviews were carried out in a private room where only the interviewer and one participant were present at the time of every interview. Interviews lasted an average of 45 min and were conducted in English which is the language of instruction in the Kenyan education system. In a few instances, participants responded in Kiswahili. All interviews were audio-recorded, transcribed verbatim and translated to English as needed. The interviewers then randomly selected two transcripts each and read them while listening to the audio-recordings to ensure accuracy of transcription and translation.

### Data analysis and reporting

Data in from of transcripts were managed using NVivo software version 12 (QSR International). Analysis followed the qualitative descriptive approach which generates data that describe the who, what, and where of events or experiences, thereby helping researchers gain insights about a phenomenon that is poorly understood [37, 38]. The researchers constructed themes from the data through an iterative coding process based on Andersen’s behavioral model modified for vulnerable populations [30]. Coding was done independently by two members of the study team, who then compared codes for agreement until a consensus was reached on whether to merge some codes, get rid of others or come up with new ones. Themes and sub-themes were described and supported with illustrative excerpts from the interviews. Excerpts were attributed to participants using pseudonyms and age in years. Reporting of the study followed the consolidated criteria for reporting qualitative studies (COREQ) [39].

### Data trustworthiness

Four criteria (credibility, dependability, transferability and confirmability) were used to ensure trustworthiness of the data [40]. For credibility, the study used well-founded methods for qualitative investigation. Prior to data collection the interviewers had created a working rapport with the participants from prior interactions during the quantitative survey. At the data collection stage, iterative investigation was used by rephrasing previously asked questions to confirm and acquire deeper understanding of the information provided by participants from their initial responses to questions. Member checking was done “on the spot” by paraphrasing and summarizing what the participants said at intervals and at the end of each topic of discussion, respectively. To address dependability, the study processes have been reported in sufficient detail to enable future researchers reproduce the study. For transferability, details about the study methods have been provided to help comprehend and compare/contrast the findings of this study with those of similar studies. Confirmability has been catered for by supporting the findings with verbatim quotes from study participants.

### Ethical considerations

The study protocol was approved by University of the Witwatersrand Human Research Ethics Committee-Medical (Reference number. M200215) and University of Nairobi-Kenyatta National Hospital Ethics and Research Committee (Reference number. P990/12/2019). Participants provided written informed consent before taking part in the study. Participants received Kenyan shillings 1,000 (approximately $10 at the time) for travel-related expenses to and from the study site, and their time.

## Results

### Participant characteristics

The sociodemographic characteristics of study participants are summarized in Table 1.

**Table 1:** Sociodemographic characteristics of YMSM (N = 22)

Variable	Category	Frequency (%)
Age	18–20	10 (45.5)
	21–24	12 (54.5)
Gender identity	Cisgender male	22 (100.0)
Sexual orientation identity	Gay	16 (72.7)
	Bisexual	6 (27.3)
Institution attended	University	11 (50.0)
	College	11 (50.0)
Ownership of institution attended	Public	13 (59.1)
	Private	9 (40.9)
Type of course studied	Science, technology, engineering and mathematics	12 (54.5)
	Arts, business and humanities	10 (45.5)
Year of study	1st and 2nd year	13 (59.1)
	3rd and 4th year	9 (40.9)
Residence	College housing/ rented hostel	16 (72.7)
	With family	6 (27.3)
Ever attended boarding school	Yes	19 (86.4)
	No	3 (13.6)
Main source of financial support	Parents/guardians	14 (63.6)
	Employment	8 (36.4)
Had smartphone at time of study	Yes	21 (95.5)
	No	1 (4.5)

### Qualitative analysis

The following sections explore the main themes that were defined from analysis of the interviews with YMSM. The themes include: YMSM’s varying experiences depending on type of health facility, priority health needs, desired HCP characteristics and perspectives on possible role of digital health interventions in improving access and use of health services.

### Varying sexuality-based experiences depending on type of health facility

Overall, participants reported experiencing prejudice, stigma and discrimination that manifested in various ways in public and institution-based health facilities. In contrast, participants reported perceptions of being handled fairly in private health facilities, community pharmacies and MSM-friendly clinics.

### Experiences in public health facilities

Participants reported experiences of non-affirming behavior by HCPs in public facilities, and held these experiences as a reason for their dislike of these facilities. One participant recounted how a friend who had been sexually assaulted was ridiculed and victimized by a HCP when he went to seek care.

“There’s a time I took my friend, because he had been raped… and then this person (HCP) asks, “Are you sure you were raped or it was your deal gone wrong?” So you are like, “this person is traumatized, why are you even asking such questions?” “That is why I hate public hospitals by the way. I would rather die.” Bobby, 23 years.

Other unpleasant behavior in public health facilities entailed HCPs pressurizing participants to disclose having engaged in same-sex practices, then using this information to gossip with other HCPs about a client when the client was still in the health facility. One episode stood out.

“My friend had some wound in the anus. When he went to the hospital, he found a lady… but the lady was just triggering him with questions so that he may tell her the truth… she created a friendly environment so that he may come out openly… So after that, he told her it was a guy (he had sex with). So on telling her, suddenly the lady changed… she became silent then asked him some other questions that are health related then she went out. After that, another doctor came in…. she pretended as though she was looking for something… have you ever seen a person looking for something but it is your eyes they are looking at? Yeah… then she left. Another one, now a male doctor came in…the male doctor is (sic) pretending to be looking at those testing kits and such but then when he left he looked at him. Then after a while the old lady now came back… so it was just obvious that she went outside there to gossip… they (HCPs) gossiped and gossiped about him!” Dylan, 24 years.

Due to loss of privacy and confidentiality resulting from such experiences, participants became afraid of visiting the same facilities in future, even if the services they needed were not related to sexual health.

“He wasn’t taken through a very good experience… so, he will be afraid…the fear of going to that place, that those people know who I am. So, I won’t go there…even if you are going to get normal (non-sexual health-related) services, it will stop you…yeah.” Dylan, 24 years.

These experiences also caused participants to view HCPs with suspicion and mistrust, resulting to non-disclosure of same-sex practices to HCPs. Participants were afraid HCPs would share this information with other people such as participants’ neighbors, thereby negatively impacting on the well-being and safety of participants in the community.

“If I go to a health center near where I live, and I am exposed by anybody working there that I normally sleep with other men, if the information leaks to the public in the area where I am staying, it will affect me because I am normally considered to be a straight person. It will just affect my being in the area and I won’t feel safe anymore.” Damian, 21 years.

There were also instances where HCPs used what participants perceived as derogatory terms, causing discomfort to participants to an extent where participants would leave the facilities without receiving care.

‘My friend chose to go to a public hospital. So the doctor that he got was a man…so he was asked what’s wrong with him and he said he has anal warts. Then he (the doctor) asked him, “Wewe ni shoga (are you gay)?” That word (shoga) is what he told me hurt him the most because there is when your friend who is gay and they call you ‘shoga’, you will understand. But if it’s someone let’s say someone in an office and they tell you that…you will feel you are not comfortable at all. He told me he felt that he had been condemned so much…so he just left. He said he will never go back there.” Cedric, 22 years.

Participants were also highly sensitive to external cues such as the way other clients visiting the facilities looked at them, which made participants feel as if the other clients knew the participants were MSM. Such cues elicited feelings of discomfort, to an extent of participants deciding to leave the facilities without receiving care.

“In the public facilities, there is abuse, there is harassment, so even when you are on that queue the looks you get from other people you will just feel you are the odd one out. So if they know you are MSM, you’ll just have to go away.” Poppy, 22 years.

However, despite these undesirable experiences, there were instances where participants stood up for themselves, confronted the HCPs, insisted and received the services they needed at the time.

“I have had an instance where my friend was telling me how when he went to a public clinic and the doctors were kind of discriminating… because when they were treating him for an anal STI they were like, “for you to get this (STI) you must have had sex with a man so you must bring him before we treat you.” It was a terrible experience. … but he was like, “yeah this is a government facility; I should seek services here as you are supposed to give me the services.” I mean, he made it clear and they just gave him the services without having to bring his partner.” Benny, 18 years.

Though the treatment of STIs rightly requires partner tracing and treatment, it is not a mandatory requirement for the treatment of the individual seeking care. In the above instance, the HCP seemed to make it a mandatory requirement and their tone was perceived by the participant as being harsh and discriminatory.

### Experiences in institution-based health facilities (campus clinics)

Though participants noted that the institution-based health facilities (campus clinics) were open for all students, they felt these clinics did not focus on their needs such as management of anal STIs. If and when they visited these clinics, they had to conceal their same-sex practices from the HCPs so as to be able to receive care and avoid ill treatment by the HCPs. If they felt they had to disclose their same-sex practices to the HCPs, then they would rather not visit these clinics.

“Our facility is accessed by everyone, like it’s not specifically for MSMs. When you go there, you will have to go like any other person. With your secret hidden so far, you will get very good services and no one will treat you awfully. But when you slip and go and say, I am gay… or you have some symptoms related to being gay like an STI in your ass… the environment won’t be safe for you anymore because people will be looking at you with those burning (hostile) eyes.” Harley, 21 years.

“It (campus clinic) doesn’t focus on people like me, LGBTs, you know… So when you are going to tell the nurse some sensitive information it’s like you are exposing yourself, so I would rather stay like that (without seeking care). I can’t, I can’t risk.” Jones, 22 years.

To support the lack of focus on MSM health needs, one participant narrated how his friend was not able to obtain treatment for anal warts. Though the nurse told the friend they did not treat such conditions, the perceived dismissive way in which he was handled by the nurse made him believe it was a denial and not lack of service.

“I have a friend… he belongs to the community (MSM). So, he went to seek for services at the school hospital. He was having an MSM issue… anal warts. So, when the nurse found out how it happened, she told him they don’t deal with STIs of such nature. She told him maybe he seeks treatment outside school. And it’s not that they (HCPs) can’t (treat anal warts) … it is just because of the fact that he is from the community (MSM). He didn’t even get to see the doctor. He was just dismissed verbally without anything… it was like an entire scene.” Soy, 20 years.

For these reasons, participants sought services for general health needs from campus clinics but if they had a sexual health need, then they opted to visit MSM-friendly clinics.

“If I have let’s say a cough, stomach upset, I usually go to the clinic in campus… but if maybe I have an STI, I won’t go there… I will go to the clinics that support MSM.” Dylan, 24.

### Experiences in private health facilities

Participants who had medical insurance or who could afford to pay out of pocket, opted to seek care from private facilities and community pharmacies where service provision is for profit. Participants reported that HCPs in these facilities did not discriminate, express judgmental attitudes nor gossip about their clients. Participants also perceived services offered in these facilities to be of high quality.

“I have an insurance cover under my parents. If I am sick, I go to a branch of a private hospital near where I stay. Their services are excellent. They don’t discriminate. I really like the place.” Soy, 20 years.

“If it is a private clinic where you have to pay, no one will ever judge you… I mean your money talks. You pay and you are like if they start gossiping you go find another clinic. So if it is private clinics it’s so good.” Benny, 18 years.

“If you are good (have money), you can go to the chemist (community pharmacy), explain your problem and they will sort you out… as long as you are paying, they have no problem.” Damian, 21 years.

### Experiences in MSM-friendly clinics

In MSM-friendly clinics, participants found a reprieve from the negative experiences in public facilities and campus clinics, and the cost implication of seeking services from private facilities and community pharmacies. HCPs in MSM-friendly clinics were perceived as non-judgmental and services were offered at no cost to clients.

“As per now, MSM are very aware of the friendly facilities, more so my friends… because even I, it is a friend who told me. He told me, “by the way, if you ever want anything, anything like STI treatment or these commodities… lubes, condoms – there’s a place where you can get them without being asked anything”. And I was very interested because before that, I was very afraid… so even though like you have not yet felt unwell but you would ask yourself, what if I get sick? where shall I go?” – Dylan, 24 years.

“If I had an STI, I would go to the MSM facilities since it is a non-judgmental place… and a friendlier place… also, you can be treated for free, there are no costs.” Damian, 21 years.

To sum it up, the unpleasant experiences in the hands of HCPs in public health facilities and campus clinics, and the ensuing mistrust of HCPs kept away some participants from seeking services, even at MSM-friendly clinics. This in turn led to poor health outcomes.

**‘**If you go to a public hospital or campus clinic and get a bad experience, there’s that trust that someone loses. They (YMSM) can’t trust anyone. So, it’s hard for them. You have to convince them too much… I’ve had friends who have waited until their warts have become really bad because they think, even if I go to an MSM-friendly clinic, how are they going to see me?” Bobby, 23 years.

### Priority health needs

Participants identified mental health, HIV/STIs and substance abuse prevention and control services as their priority health needs. They strongly expressed the need for mental health services, especially counseling services to help them cope with various life situations that they were experiencing.

“For me, mental health should be like the first priority. We need to have a lot of counselors because people are going through a lot in campus. Some people it’s struggling with accepting themselves as gay, some it’s their family not accepting them, some have health problems, money, peer pressure, break ups… and all this stuff which you know can lead to depression.” Benny, 18 years.

For HIV/STIs, due to high sexual activity, participants felt that they needed health education on various prevention strategies such as pre-exposure prophylaxis (PrEP), post-exposure prophylaxis (PEP) and condoms, and control strategies such as HIV self-testing and screening for STIs.

“The health facilities should reach out to educate us on PrEP, PEP, on use of condoms, lubricants and self-test kits (for HIV). We also need regular screening for STIs because as young adults we are very sexually active.” Jamie, 21 years.

There was also need to address issues of substance abuse since participants noted that this was rampant among them, mostly due to peer pressure and an emerging trend of sexualized substance use.

**“**Yeah…because we are a small community I would say the influence (to use drugs) is infectious… there is a lot of peer pressure… you ask yourself, everyone does it, why not me? That’s what people say… it is now becoming a culture because most people can’t have sex without using drugs… weed (marijuana), alcohol, cigarettes… all those. If we could have services that would help out with these issues, that would be really great.” Ayan, 21 years.

### Desired HCP characteristics

Participants were asked to describe the characteristics of their preferred HCP. Responses showed that participants were more interested in their HCP’s inter-personal skillset than in demographic characteristics such as gender, sex and sexual orientation. For instance, participants wanted a HCP who could create a good rapport with them, communicate effectively and be willing to help, over needing the HCP to be of the same sexual orientation as the participant.

“The doctor doesn’t have to be LGBTQ. He or she just has to be open-minded, have the heart of helping… like be passionate about what they do and attend to everyone no matter their sexuality.” Aiden, 20 years.

“I think they (HCPs) should be very professional and have that rapport with us, and always be there to help and guide us…… so we feel like we are talking to a friend. They (HCPs) should know how to communicate with us young adults and make us feel comfortable expressing ourselves, no matter our sexuality.” Jamie, 21 years.

Participants also expressed a desire to have HCPs who are knowledgeable about the reality of MSM being part of society, MSM’s unique health needs, and able to provide care to MSM without discrimination.

“I think all doctors should go through a program that teaches them that there are these kind of people (MSM) and this is how you are supposed to treat them, and they are actually normal people… it’s not like a case out of the ordinary.” Benny, 18 years.

“They (HCPs) should know that there are those diseases like anal warts that mostly affect MSMs and they should help them… even if they’re queer, do not discriminate, just give them the services they are looking for.” Cedric, 22 years.

Noteworthy, participants pointed out the need for HCPs to avoid judgmental attitudes and gossiping about clients, as well as respect clients’ privacy. That way, the health facility climate would be conducive for not only MSM clients, but for everyone seeking care.

“They should stop judging people, stop talking about people and respect people’s privacy. That way, the whole healthcare system will be good for everyone, not just gay people.” Jones, 22.

### Potential role of digital interventions in improving access to and use of health services

Participants offered various suggestions on how digital interventions could help improve access to and use of health services by them. Participants noted that, unlike traditional educational materials such as pamphlets and brochures, social media is more appealing to use, has the capacity to reach more people and could be used as a discreet platform to advertise HCPs’ services and provide health education on various sexual health topics such as PrEP and condom use.

“I have seen these online posters that you make… pretty posters that MSM will know are meant for them but not the other people… so when you send them out there, they (MSM) will repost on their status (on social media platforms) so people will have the information on where they can access the services… and it will spread fast.” Jones, 22 years.

“Most people nowadays don’t have the time to read. You cannot give them like a pamphlet on PrEP to go read. They will just trash it in the next dustbin because also they do not want to be seen with it. If the clinics can share short videos on Instagram or YouTube on how to like properly use condoms… the benefits of PrEP… one can watch the videos privately and the videos can go viral and reach most of the MSM people and educate them.” Jamie, 21 years.

Participants also felt that mobile phone applications that provide for online consultation with a HCP, could help YMSM access services in a non-judgmental way since the nature of the online HCP-patient interaction is anonymous.

“And then there are the online doctors. They can give you services if you don’t like going to a facility where you might be judged. The good thing is that the internet never judges you.” Myles, 19 years.

Compared to in-person purchasing at the community pharmacies, online shopping also presented a discreet and convenient way to purchase health commodities such as condoms and lubricants.

“You know people fear to go out to chemists (community pharmacies) and buy condoms and lube… so you can go to apps like Mydawa and order then they bring at your doorstep or any other place you agree. So the internet really helps in that way.” Harley, 21 years.

Participants also felt that interactions between MSM on MSM-focused geosocial networking applications such as Grindr could facilitate locating facilities that offer health services such as HIV/STIs testing.

“There’s an app called Grindr… people are always like I last tested (for HIV) on 1st May, so you would be like, “where did you test” … so they mention clinic X and they are like “where is it?” I say it is in town… so even if you don’t get to meet, the person will know there is a clinic in town where they can get tested.” Aiden, 20 years.

Participants observed that online platforms could also be useful in giving HCPs anonymous feedback that HCPs could use to improve service delivery.

“If there is a way a clinic has a platform online where it doesn’t reveal who texted this or that thing, yeah it can be good because they (clients) will give out their grievances and that will help the clinic to improve the services that they provide.” Cedric, 22 years.

Lastly, participants noted that meeting peers online, sharing experiences with and learning from them how to navigate various life issues, was beneficial in offering social support for participants overall well-being.

“Meeting other people online who are like you, who are going through the same problems like you, and sharing the experiences of how they dealt with it, and knowing you are not alone, and you will overcome, is really helpful.” Benny, 18 years.

## Discussion

This study provides numerous insights about healthcare engagement among YMSM in Nairobi, Kenya. We found out that YMSM experienced prejudice, stigma and discrimination in public and institution-based health facilities, but were handled fairly in community pharmacies, private and MSM-friendly health facilities. YMSM mainly sought services for sexual health needs, but also mentioned needing mental health services as well as help with issues related to substance abuse. YMSM expressed a desire to have HCPs who were knowledgeable about their unique sexual health needs, and willing to offer services in a non-judgmental way. The discreet nature, appeal and potential of digital media to reach more people compared to traditional media, was considered a collective strength that could be leveraged on to improve access to and use of health services by YMSM. Except for experiences of stigma and discrimination by HCPs, health needs, desired provider characteristics and perspectives on digital health interventions expressed by YMSM in our study, were somewhat similar to those identified in a qualitative study among older heterosexual Malaysian men [41].

Consistent with Andersen’s model modified for vulnerable populations [30], our study found out that for YMSM, health services use was largely affected by factors such as societal stigma and discrimination against sexual minority people, which manifested in the way HCPs in public and institution-based health facilities handled YMSM. To navigate the unfair treatment by HCPs, participants used the public and institution-based health facilities for general health needs but visited community pharmacies, private and MSM-friendly health facilities when they needed sexual health services. This coping strategy has also been observed among YMSM in the USA [42]. Despite its benefits as observed in this study, the practice of separating healthcare seeking may not serve the best interest for the overall health and well-being of YMSM. Ideally, like other students, YMSM should be able to access the services they need from their campus clinics, and be referred appropriately when the services are not available. Seeking sexual health services outside their campuses has cost implications arising from needing to have medical insurance or paying out of pocket in private health facilities and/or community pharmacies. More than a third (34.3%) of Kenyans live below the poverty line [43], and only a minority of Kenyans (11%) are covered by the most affordable public health insurance scheme [44], with both scenarios likely replicated among the YMSM community. Even when the services are offered free of charge in MSM-friendly clinics, YMSM must allocate time and incur transport costs to get to and from these clinics, given that there is only a handful of such clinics in the Nairobi metropolis. We therefore suggest training and sensitizing HCPs working in the campus clinics on the unique health needs of YMSM, and how to provide the latter with culturally competent services in a non-judgmental way, and thus offset the costs and inconveniences borne by YMSM when seeking services externally. Previous sensitization and skills training for HCPs in Kenya has indeed been shown to improve service provision for MSM clients [45].

The findings from this study have implications for clinical practice and training. Previous studies have demonstrated the importance of MSM disclosing same-sex practices to HCPs as this may facilitate appropriate consultation including risk assessment for HIV/STIs [46, 47]. In the current study, participants often spoke of experiencing challenges in healthcare settings when they sought treatment for anal STIs, which were of special interest to them based on their sexual behaviors. Disclosure of same-sex practices whether voluntary or through coercion by HCPs generated stigma and discrimination which manifested in various ways including verbal insults, gossip and denial of services, as has been observed in other settings in sub-Saharan Africa [48–53]. This finding further reinforces the need for training and sensitizing HCPs in the public and institution-based health facilities on the provision of comprehensive, affirming and equitable services for YMSM. Since Kenya has a dedicated national program that leads the HIV response among key populations (including MSM) [19], we propose that tertiary institutions piggyback on this program to provide such training and sensitization to their HCPs. Through the key populations program, the ministry of health and other stakeholders should also offer similar training and sensitization to HCPs in public health facilities. In addition to imparting knowledge on the health needs of MSM, training should also seek to equip HCPs with skills and attributes that participants desired them to have such as effective communication, empathy and a willingness to help. There is also the opportunity to make the training much more encompassing so as to cover not only sexual health services but also other participant-identified priority health needs, including mental health, and substance abuse prevention and control services. The scope of training should also possibly be extended to cover the health needs of other sexual and gender minority youth. Previous work done in Kenya found out that sexual health counseling by HCPs commonly addresses only heterosexual behavior, partly because training curricula do not include issues around same-sex practices [54]. The reported behavior of HCPs in our study possibly lends credence to this earlier finding. As a result, we propose that training curricula for health professions students should include modules on sexual and gender minority health so that as the students transition to becoming HCPs, they have already been introduced to the topic and this could serve as a foundation for further capacity building.

This study also highlights the potential role digital interventions could play in improving health services access and use by YMSM. Participants observed that digital platforms could allow them to circumvent some of the barriers they encounter while seeking services in physical health facilities, especially the experiences of stigma and discrimination, as has been highlighted in a previous commentary [55]. The use of digital platforms could also confer other benefits such as allowing YMSM to purchase health commodities (lubricants and condoms) anonymously and interact with their peers for social support. The appeal of digital media is pertinent in ensuring that information contained in such media reaches a large audience, and this could be helpful for HCPs seeking to advertise or offer their services online. This is particularly important since YMSM joining the tertiary institutions especially from areas outside the city may not be familiar with places where they can access MSM-friendly services, and may also be less aware of what constitutes behaviors that may put them at risk for various health conditions such as HIV/STIs, mental health illness and substance abuse. Digital platforms could also be useful for users in giving feedback to HCPs that could help improve service provision. Work done by researchers in Kenya has demonstrated that tertiary students are regular and savvy users of the internet, with approximately 95% owning smart phones [56]. Accordingly, HCPs should seek to meet YMSM where they are – online. As seen from our sample, 95.5% of participants owned a smart phone at the time of the study. Previous research has demonstrated the usefulness of digital interventions in addressing various health challenges such as HIV/STIs [57–59], mental health [60] and substance use [61] among YMSM, as well as accessing social support in coming out and finding a community to belong to [62]. An ongoing study in Kenya is assessing acceptability, feasibility and cost implications of online PrEP and PEP delivery among a diverse clientele within the Nairobi metropolis [63]. We suggest that digital interventions should be considered for service delivery among YMSM in Kenya, given the demonstrated potential usefulness of such interventions [64], and the ubiquitous use of smart phones in this population.

As noted by the participants, the hostile health climates in public health facilities and campus clinics caused YMSM to postpone healthcare seeking until symptoms worsened, resulting in poor health outcomes. Additionally, ill treatment by HCPs in these facilities caused some YMSM to distrust the healthcare system as a whole and become skeptical about seeking services even from the MSM-friendly clinics. Perhaps the HCPs who exhibited stigma and discrimination were emboldened to treat YMSM with partiality due to the criminalization of same-sex practices through the colonial Kenyan penal code [11]. Nevertheless, the Constitution of Kenya guarantees every Kenyan citizen the right to the highest attainable standard of health [65]. HCPs as duty bearers need to be made aware and/or reminded of this right as they have a duty to ensure the enjoyment of this right by everyone, including YMSM. As observed from the findings, one YMSM was able to demand for and receive treatment for an anal STI in a public health facility. Subsequently, interventions to increase the agency of YMSM to demand for this right are also requisite.

One of the strengths of this study is that it was conducted in the capital Nairobi which has a range of healthcare settings including community pharmacies, public, private and MSM-friendly health facilities. Therefore, it was possible to obtain and compare information on healthcare engagement experiences from each of these settings hence increasing the robustness of the study. Nevertheless, this study is not devoid of limitations. For example, the findings may not be representative of other urban, and indeed semi-urban and rural areas in the country with different settings. In addition, the study recruited participants from a larger sample that had previously been engaged in health research. As such, the views expressed here may not reflect those of participants without prior research engagement. We mitigated this potential source of bias by asking participants to share their own healthcare engagement experiences, as well as those of their friends, who may have or not engaged with research beforehand. Even so, we note that participants predominantly shared their friends’ experiences and whereas this could be a true reflection of what indeed happened, it is possible that due to social desirability bias, some of the experiences shared as coming from friends could actually have been participants’ own experiences. Despite these limitations, the study adds to the scarce body of knowledge on healthcare engagement among YMSM in sub-Saharan Africa in general and Kenya in particular. These findings can be used to inform the design and development of interventions to improve access to and use of health services by YMSM, and inform the design of similar studies in future.

## Conclusion

YMSM in this study reported that healthcare services in public and institution-based health facilities were more prejudicial, stigmatizing and discriminatory compared to those available at community pharmacies, private and MSM-friendly health facilities. To address these health disparities experienced by YMSM, there is urgent need to train and sensitize HCPs in public and institution-based health facilities so as to equip them with knowledge and skills required to offer culturally competent services that meet the unique needs of YMSM. Digital health interventions should be considered for reaching YMSM with the services they need, including but not limited to sexual and mental health services.

## Data Availability

The datasets generated and analyzed during the current study are not publicly available since they are qualitative interviews with potentially identifiable information from members of both a criminalized and marginalized population. However, the datasets are available from the corresponding author on reasonable request.

## Abbreviations

HCP: healthcare provider
HIV: human immuno-deficiency virus
MSM: men who have sex with men
PEP: post-exposure prophylaxis
PrEP: pre-exposure prophylaxis
RDS: respondent-driven sampling
STI: sexually transmitted infection
YMSM: young men who have sex with men
